# A cost-effectiveness analysis model of Preventicus atrial fibrillation screening from the point of view of statutory health insurance in Germany

**DOI:** 10.1186/s13561-020-00274-z

**Published:** 2020-06-09

**Authors:** Ralf Birkemeyer, Alfred Müller, Steffen Wahler, Johann-Matthias von der Schulenburg

**Affiliations:** 1 Ulm Herzklinik, Krankenhausstraße 5, 89231 Ulm, Germany; 2 Analytic Services GmbH, Jahnstr. 34c, 80469 Munich, Germany; 3 St. Bernward GmbH, Friedrich-Kirsten-Straße 40, 22391 Hamburg, Germany; 4 Universität Hannover, Welfengarten 1, 30167 Hannover, Germany

**Keywords:** Atrial fibrillation, Screening, Prevention of stroke, Cost-effectiveness analysis

## Abstract

**Background:**

With atrial fibrillation (AF) the risk of stroke is 4.2-fold increased to a comparable population without AF. This risk decreases by up to 70% if AF is detected early enough and effective stroke preventive measures are taken as recommended by international guidelines. Long-term studies found large number of subjects with undiagnosed AF. Preventicus Heartbeats” is a hands-on screening tool for use on smartphone to diagnose AF with high sensitivity and specificity. The aim of this study is to research the cost-effectiveness of systematic screening for AF with this smartphone application.

**Method:**

Employing a Markov model we analysed the cost-effectiveness of the “Preventicus Heartbeats” screening for Germany, i.e. from the perspective of German statutory sick funds.

**Results:**

For a cohort of 10,000 insured 75-year-old the use of the diagnostic app could avoid 60 strokes in the remaining lifetime thereof 32 strokes in the next four years. Former models have applied similar cohorts. The same cohort showed an increase in quality-adjusted life years (QALY) in the remaining lifetime of 165 QALYs in the scenario with screening versus.

without screening and a decrease in discounted lifetime costs (including risk compensation effects) of €129 per participant (€148 for male, €114 for female participants).

**Conclusions:**

The modelling demonstrates the health benefits and economic effects of an implementation of a systematic screening on AF with “Preventicus Heartbeats”, given the perspective of the German payer, the statutory health care system.

## Background

Annual costs of acute and follow-up treatment of strokes were € 6.5 billion in 2015 in the German health care system. Thereof ischemic strokes alone accounted for € 5.1 billion [1]. One hundred six thousand persons a year insured in German statutory sick funds suffer a first-time stroke and 66,000 insured persons a recurrent stroke [2]. Based on the Erlangen registry [3], calculated lifetime costs of a stroke are around € 43,000. Epidemiological studies also noted that - mainly due to German demography - the lifetime prevalence has risen significantly since 1998 [4].

Atrial fibrillation (AF) is the most common arrhythmia of clinical significance [5]. It is a supraventricular tachyarrhythmia with uncoordinated activity of the atria and frequencies between 350 to 600 bpm. Result is functional loss of activity of the atria with reduction of cardiac output [6]. AF is associated with increased morbidity, especially stroke and heart failure, and increased mortality [7–10] and constitutes a significant public health problem [11–13]. The prevalence of diagnosed AF is estimated 1% in Germany with increase in the old age (8% in population above 80 years) [14].

Mainly discovered is AF in patients who seek medical treatment due to related clinical symptoms (palpitations, shortness of breath, etc.) or in previously asymptomatic patients after they have suffered a stroke which was possibly caused by cerebral embolism [5]. Due to the relatively short observation periods, e.g. only around 60 s with a usual resting ECG, some screening studies provide low detection rates of previously undetected AF.

The Swedish “Strokestop” study [15] with 75-year-old subjects discovered a previously undetected AF in 3.0% of subjects using phased ECG recordings during a two-week measurement. The presence of atrial fibrillation was already known in 9.3% of the subjects. Various similar studies aimed at the identification of previously undetected AF and reported a ratio of unknown to known AF between 20 and 45% increasing with age [16, 17].

Analyses of long-term data released in the 1980s and 1990s showed a significantly increased likelihood of a stroke in patients with AF [8]. It was demonstrated that in patients with atrial fibrillation the probability of an ischemic stroke was increased 4- to 5-fold, but also AF is associated with higher mortality, and, if stroke occurs, AF patients suffer a significantly higher degree of disability, death and risk of a second stroke within 12 months compared to non-AF patients [18–22].

Anticoagulating agents to reduce the risk of stroke with AF have been in clinical use since the 1980s. Several studies found oral anticoagulation to reduce the risk of stroke by 65–80% in patients with AF [23, 24]. Guidelines therefore require mandatory prevention with anticoagulants in AF patients with additional risk factors [25]. Vitamin K antagonists and antiplatelet agents have been increasingly replaced by direct (or “non-vitamin K antagonist”) oral anticoagulants (NOAC) in the last 5 years. They show a slightly improved effectiveness and a significantly improved safety profile compared with vitamin K antagonists, particularly with regard to bleeding [26–30].

Thus, systematically undetected AF is a systematic risk for stroke for patients who could otherwise benefit from an anticoagulation therapy. Therefore, early detection and appropriate measurements reduce the number and burden of strokes.

“Preventicus Heartbeats” is a Class 2a medical app with the purpose to detect and record the presence or absence of AF episodes by means of regular short measurements on the participant’s mobile phone. The technology is based on recordings of photopethysmographic (PPG) signals which is widespreadly used for pulse detection. By simply putting a finger on the smartphone camera the pulse curve is recorded and automatically analysed. Pathological reports are reviewed by a telecare centre before indicating the result to the user. A training programme on how to perform measurements is integrated in the app as well as aids and feedback tools. Sensitivity and specifity of atrial fibrillation detection compared to the gold standard electrocardiogram were determined in prospective validation studies [31, 32]. Participants diagnosed with absolute arrhythmia during the “AF screening” will undergo a validation phase of up to 2 weeks. A continuously recording, telemetric chest ECG event recorder (“AF confirmation”) allows the final diagnosis and an appropriate treatment of AF according to the guidelines, by ruling out incorrect screening results or results that are not relevant for treatment, which may arise from short-term arrhythmia episodes during the mobile phone measurement.

## Methods

To assess the cost-effectiveness, we employed a model to compare the use of the “Preventicus Heartbeats” screening with a scenario without screening. Financial effects of the German morbidity-oriented risk structure compensation scheme (RSC) were optionally included. The course of the health state of the screening cohort was modelled based on parameters from official statistics and publications about topics “stroke”, “atrial fibrillation and stroke” and “share of undetected atrial fibrillation”.

**Fig. 1:**
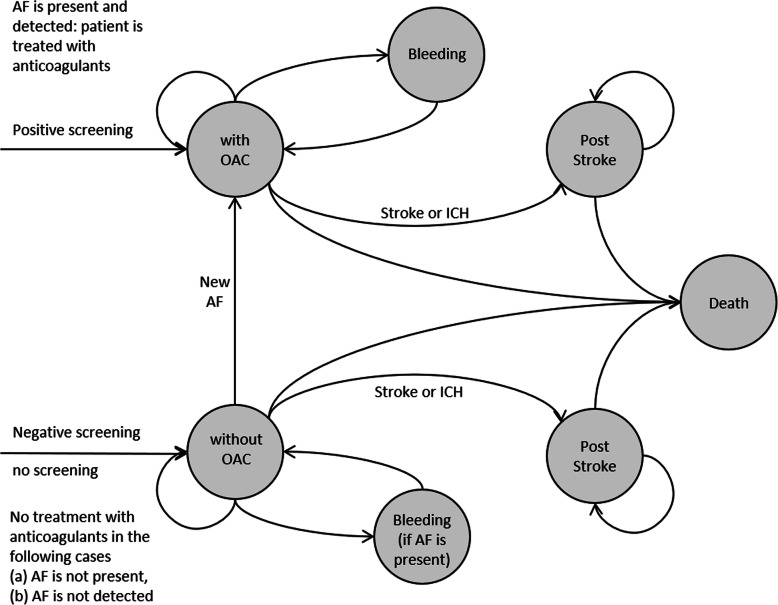
Markov model structure

The model is firstly constructed as long-term health history of a screening cohort over several periods for the remaining lifetime of the single participants. A Markov model was implemented, calculating specific states at any given point in time corresponding to different degrees of health or illness, see Fig. 1.

Depending on the events in a defined period (e.g. bleeding, stroke, death), the health state changes in the next period. This approach is a standard tool for the assessment of long-term cost-effectiveness (see [33], p. 295 et seq [34]).

Transition probabilities were defined for the transition between the health states, in which further information is included, depending on the previous health state. Age, gender, the presence of AF, treatment with oral anticoagulants and previous strokes or cerebral haemorrhages were incorporated.

In the scenario with screening, the presence or absence of AF and the quality of the screening (sensitivity and specificity of the procedure) influence the transition probabilities. In the scenario without screening, a distinction is made between presence or absence of AF. For each of the observed cases a sub-model with the different transition probabilities was constructed. The design of the model is a cohort model. The cohort is homogeneous in terms of starting age and gender.

Included are the costs of screening and the cost of anticoagulation among participants with newly detected AF and participants in whom AF was detected during the relevant time period without prior screening. During the further course of the model, the participants may suffer a stroke, cerebral haemorrhages or bleeding in other locations. The costs of these events are also included in the financial endpoint.

For additionally identified AF patients who are receiving anticoagulation treatment statutory sick funds are compensated according to the morbidity-oriented risk structure compensation scheme (RSC) if this is documented in two subsequent quarters. Similarly, strokes and cerebral haemorrhages also are compensated by RSC compensation if the patients are treated in hospital.

### Base case analysis

**Fig. 2:**
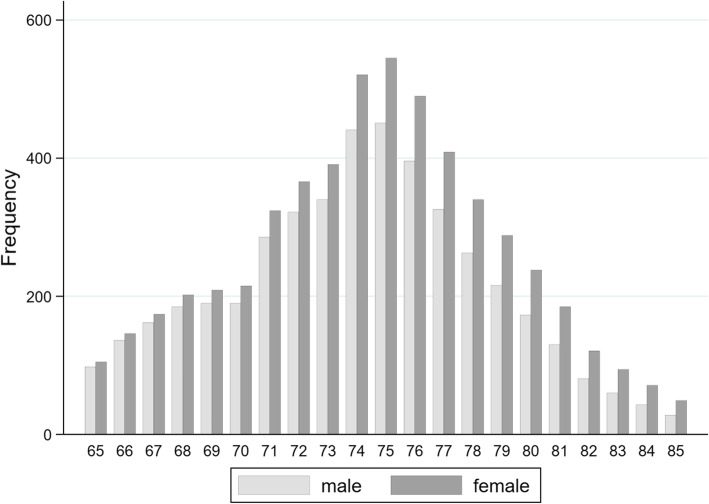
Simulated population by age and gender

Effectiveness of the screening is measured by the number of strokes prevented in participants with AF. For compatibility with the Scandinavian and Anglo-Saxon literature on the cost-effectiveness of screening measures, the effect of the screening measure is also calculated on the quality-adjusted life years (QALY) (for the QALY concept see [35]). Endpoints were calculated for a specified starting age and alternatively for men and women. The default starting age was set 75 years old in the base case, analogous to the Swedish model based on the “Strokestop” study [36]. The screening cohort in “Strokestop” was composed of insured persons (a) between the ages of 65 and 85 or (b) between the ages of 55 and 65, but who are at increased risk of AF or stroke due to underlying diseases such as high blood pressure or diabetes. The base case was calculated separately for men and women for all starting ages between 65 and 85. The events were weighted by age and gender with fictitious shares in a screening population, which included the demographic population shares. It was also assumed that the participation rate will increase up to the age of 75 and then fall (see Fig. 2). The events were weighted by age and gender with fictitious shares in a screening population, which included the demographic population shares. It was also assumed that the participation rate will increase up to the age of 75 and then fall.

In order to gain insights into the effects of the screening for an entire population, the Markov cohort model was run separately for 42 strata (men and women and all starting ages between 65 and 85 years). The strata results were then weighted by their proportions in the fictitious population.

Under the German provisions of special care (Art. 140a (2) SGB [German Social Act] V) the cost-effectiveness of any measure must be tested after 4 years.

### Deterministic sensitivity analyses

The model parameters were subjected to a series of sensitivity analyses. Here, the base case model result (no deterioration of the result was shown when the RSC compensation payments were considered) proved to be robust against the changes in the parameters. Changes to the stroke rates and costs, the amounts of the RSC compensation payments, the starting age of the patients and the prevalence of undetected AF in the screening population had the strongest influence on the result of the model.

Sensitivity analyses are used to assess the dependency of the model results on certain assumptions and the stability of the model results. Nineteen model parameters were selected, which were expected to have a stronger impact on the model result, or which had no or only inaccurate prior information when selecting the parameters for the base case. Table 6 lists the parameters for the sensitivity analysis and the selected ranges of the parameters.

### Probabilistic sensitivity analysis

While the deterministic sensitivity analysis only changes one parameter at a time and leaves the rest of the parameters unchanged, the probabilistic sensitivity analysis simultaneously changes a given set of parameters. For each of the varied parameters distribution assumptions are postulated, which either focus on the sampling error of the survey or represent further assumptions. For the analysis presented here, distribution assumptions were made for 32 parameters (see Appendix, Table 3). The distribution assumptions relate to transition probabilities, stroke costs and quality of life. This is based on the simplistic assumption of independent distributions of the parameters.

In the probabilistic sensitivity analysis, the decision problem is considered from a Bayesian point of view. The a priori distributions of the parameters are linked to the empirical distributions of the observed populations using a Monte Carlo simulation. The result is an approximate value for the a posteriori distribution of the expected values. What is presented is the result of 1000 random selections from the distributions of the 32 parameters considered to be random.

A detailed description of the model parameters used in the base case and the sensitivity analyses is given in the Appendix.

### Software

TreeAge Pro®, version 2019.2.1 was used to create and to calculate the model, including the probabilistic sensitivity analyses and the positioning of the observed alternatives about their incremental cost-effectiveness.

## Results

### Base case

#### Timeframe: remaining lifetime

**Fig. 3:**
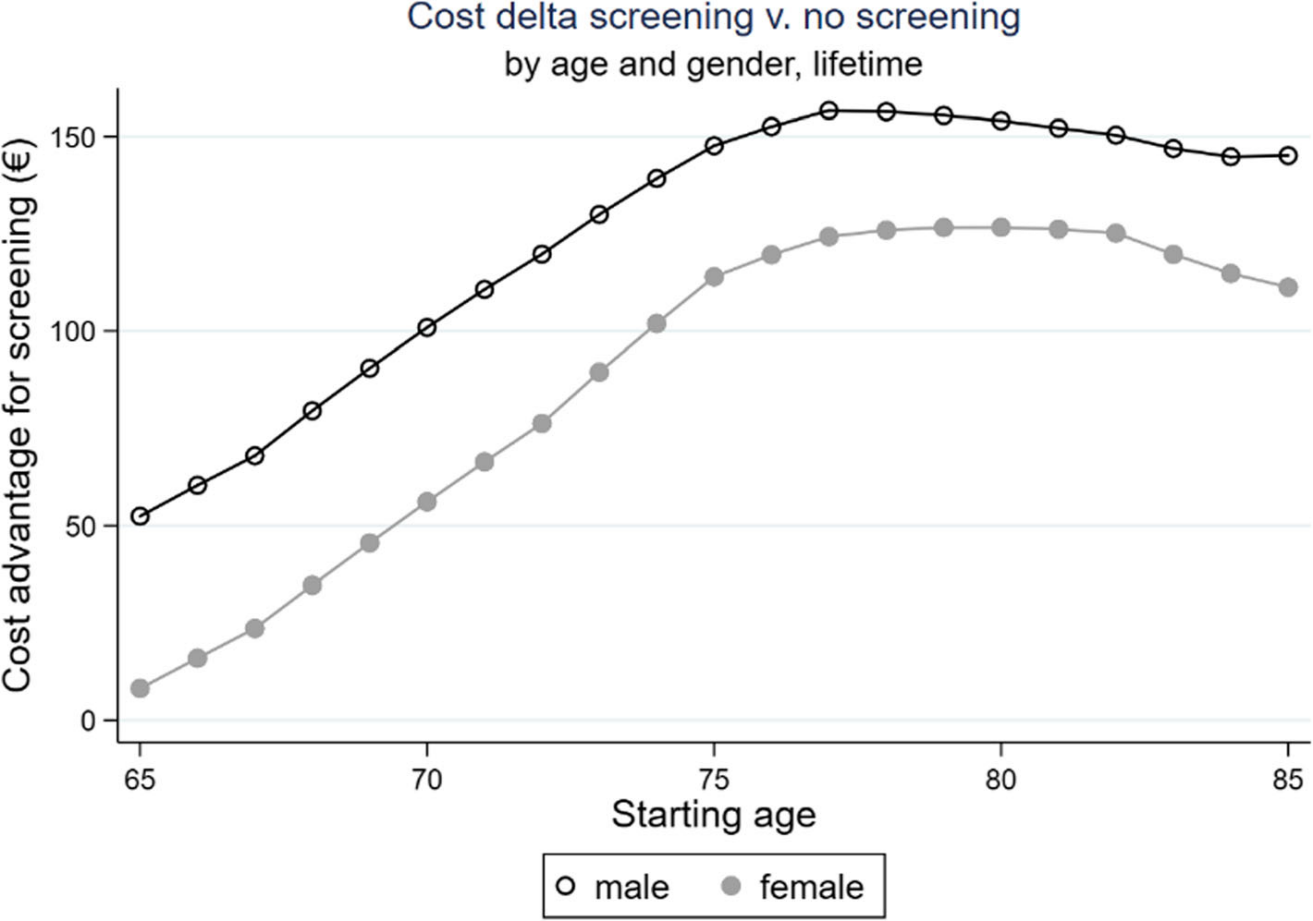
Cost delta screening vs. no screening, timeframe: remaining lifetime

The cohort with offer to participate in “Preventicus Heartbeats” screening showed cost advantage for both men and women. The cost advantage initially increases with the starting age up to a maximum, 77 years for men and 80 years for women. A higher cost advantage is found for men, due to the higher prevalence and incidence of stroke (see Fig. 3).

Including the compensation by the risk structure compensation scheme (RSC), the entire cohort outlined in Fig. 2 results in a cost advantage of €109.96 per included insured person for the “Preventicus Heartbeats” screening. This effect is approx. €35 higher in men (with €128.80) than in women (€94.43).

#### QALY delta

**F**or all subgroups observed, there is a slight increase in quality-adjusted life years. The QALY effect is correlated to starting age: men: 65 years old: + 0.008 QALY, 75 years old: + 0.018 QALY, 85 years old: + 0.021 QALY. In average the screening cohort gains + 0.015 QALYs over remaining lifetime.

#### Strokes prevented

The AF screening prevents 54 cases of stroke in a screening population of 10,000 participants, men: 61; women: 48. Initial and subsequent events were included in the calculation. At a starting age of 75 (based on 10,000 participants) 60 (men: 67, women: 55) strokes were prevented.

#### Timeframe: four-year period

Across the screening cohort outlined in Fig. 2, there is a cost advantage of €23.59 in favour of the “Preventicus Heartbeats” screening, including the compensation by the risk structure compensation scheme (RSC). This effect is approx. €23 higher in men (with €36.13) than in women (€13.25).

**Fig. 4:**
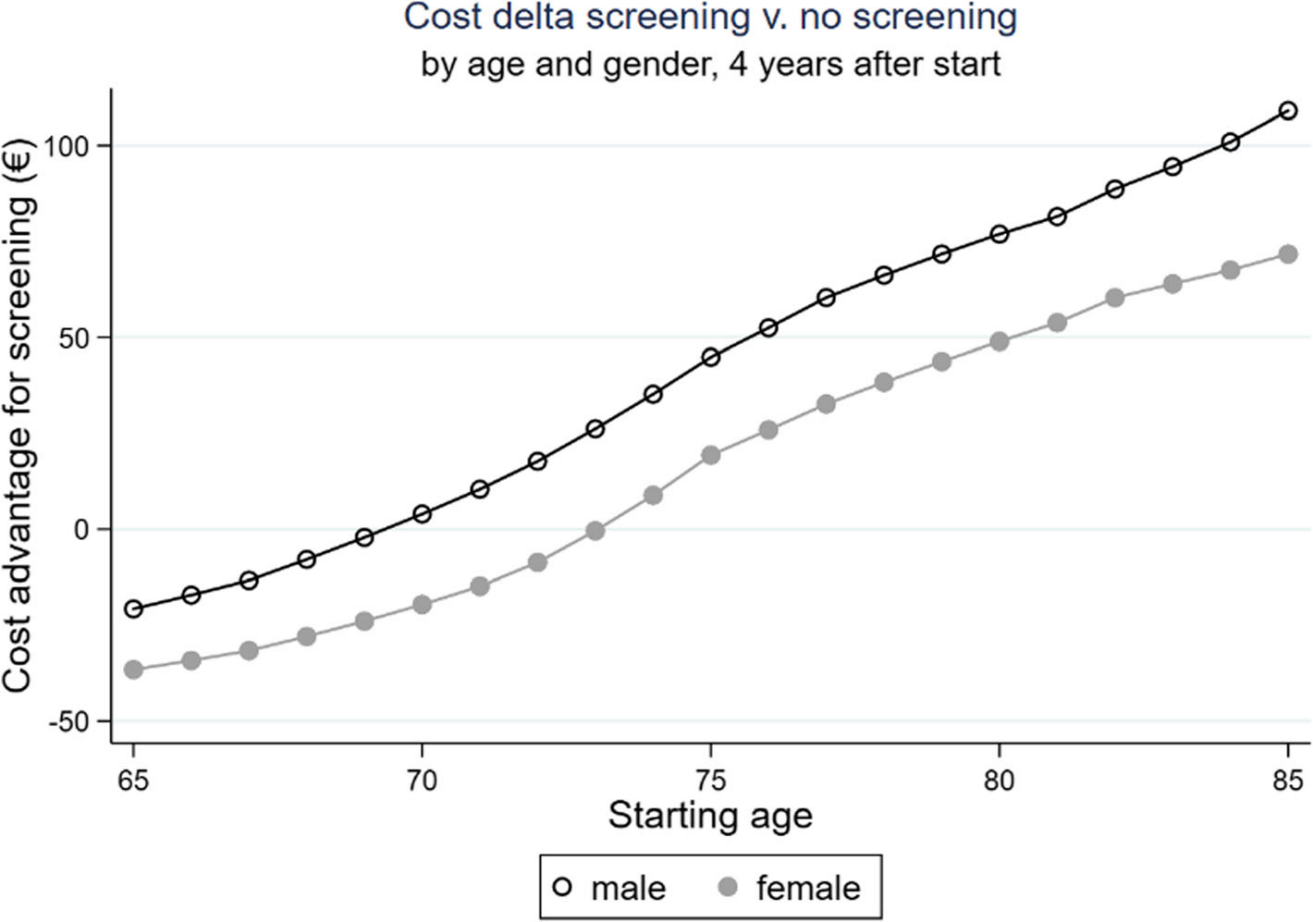
Cost delta screening vs. no screening, timeframe: 4 years after screening

Participants with a relatively low starting age (men: up to 69 years old, women: up to 73 years old) do not achieve a cost advantage (see Fig. 4).

#### QALY delta

Over the next 4 years after the start of the project, quality-adjusted life years increased by 0.002 QALY.

#### Strokes prevented

Based on the first 4 years after the screening, AF screening prevents on average 24 cases of stroke in a screening population of 10,000 participants (men: 30; women: 20). Initial and subsequent events were included in the calculation. In the shorter timeframe, at a starting age of 75 years old (based on 10,000 participants), 27 (men: 32, women: 23) strokes were prevented.

### Deterministic sensitivity analyses

**Fig. 5:**
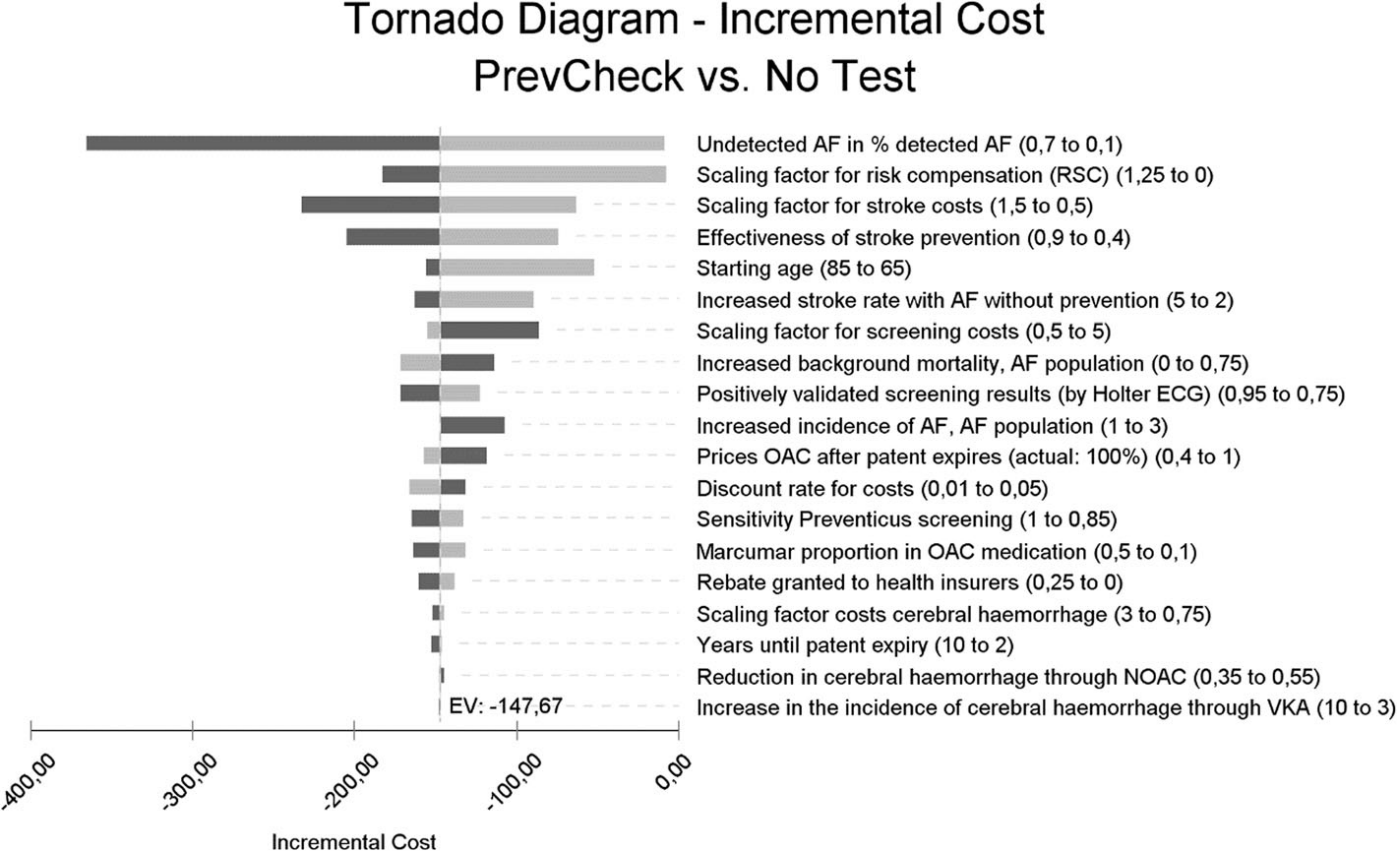
Tornado chart: effect of the isolated parameter changes of 19 model parameters on the model result (each ceteris paribus), timeframe remaining lifetime, men; base case: starting age 75 years old

Timeframe remaining lifetime: A deterministic sensitivity analysis for all selected parameters is performed in a tornado chart. The parameters are then sorted according to the fluctuation range of the endpoint (here: cost impact of the screening taking into account the effects of RSA) and presented as a deviation from a reference line (here: “EV” = “expected value”, corresponds to the base case result). In TreeAge Pro® the effects of decreased parameter values are shown in light shading and the effects of increased parameter values are shown in dark shading. Figure 5 shows the tornado chart for the timeframe “remaining lifetime” and for men with a starting age of 75. The result for women only differs slightly (Table 1).

**Table 1:** Results of the sensitivity analysis for 9 parameters with a high degree of fluctuation and the discount factor, timeframe remaining lifetime, starting age 75 years old

Parameter	Variable		Minimum value	Delta costs *	Maximum value	Delta costs a
Base case, model timefram lifetime, starting age 75 years Discounted by 3%		M F		147.67 € 113.93 €		
pAF	Undetected AF in % of detected AF population (base case: 33%)	M F	10%	8.94 € −1.18 €	70%	365.67 € 294.82 €
cStroke_ Factor	Scaling factor stroke costs 100% = base case	M F	50%	63.18 € 43.66 €	150%	232.15 € 184.20 €
RSA_Factor	Scaling factor RSA compensation payments; base case: factor 1.0	M F	0.0	7.77 € 1,99 €	1.25	182.64 € 141.91 €
orStroke_AF_ reduction	Effectiveness of stroke prevention 0% - no effect; base case: 70%	M F	40%	74.20 € 52.11 €	90%	204.81 € 161.76 €
AgeStart	Starting age of the population Base case: 75 years old	M F	65 years old	52.42 € 8.22 €	85 years old	156.65 € 126.67 €
orStroke_AF_ NOATT	Increased stroke rate with AF without prevention; Base case: factor 4.2	M F	2.0	89.75 € 64.33 €	5.0	163.29 € 127.32 €
cTest_Factor	Scaling factor screening costs 100% = base case (€47.54 / €297.50)	M F	50%	155.34 € 119.88 €	500%	86.27 € 66.28 €
orMortAF_base	Increased background mortality, AF subpop. 25% increase = base case	MF	0%	171.69 € 131.13 €	75%	113.86 € 89.36 €
Discount rate	Discount factor 3% = base case	M F	1%	164.53 € 127.87 €	5%	132.34 € 101.26 €

*a Delta costs: cost advantage for screening strategy (if positive), cost disadvantage for screening strategy (if negative)*

The model endpoint “Delta costs incl. RSA effect” is negative for all observed variants. Considering the effect of the RSA, it can be assumed that the screening has a positive earnings effect. The parameter “undetected AF in % of detected AF”, for which a relatively wide parameter range of 10% to 70% is assumed, has the strongest impact on the change in costs (in 75-year-old men: -€9 with a share of 10% up to -€366 with a share of 70% undetected AF).

The variation of influential factors linked to the frequency and costs of strokes also has a strong impact on the model result as well as the amount of the compensation payments from the RSA. In case of a complete cessation of the RSA, the model endpoint in the base case decreases from €148 to €8 for men and from €114 to €2 for women. Table 1 lists the results for 9 parameters with high fluctuation ranges.

#### Timeframe four years

**Fig. 6:**
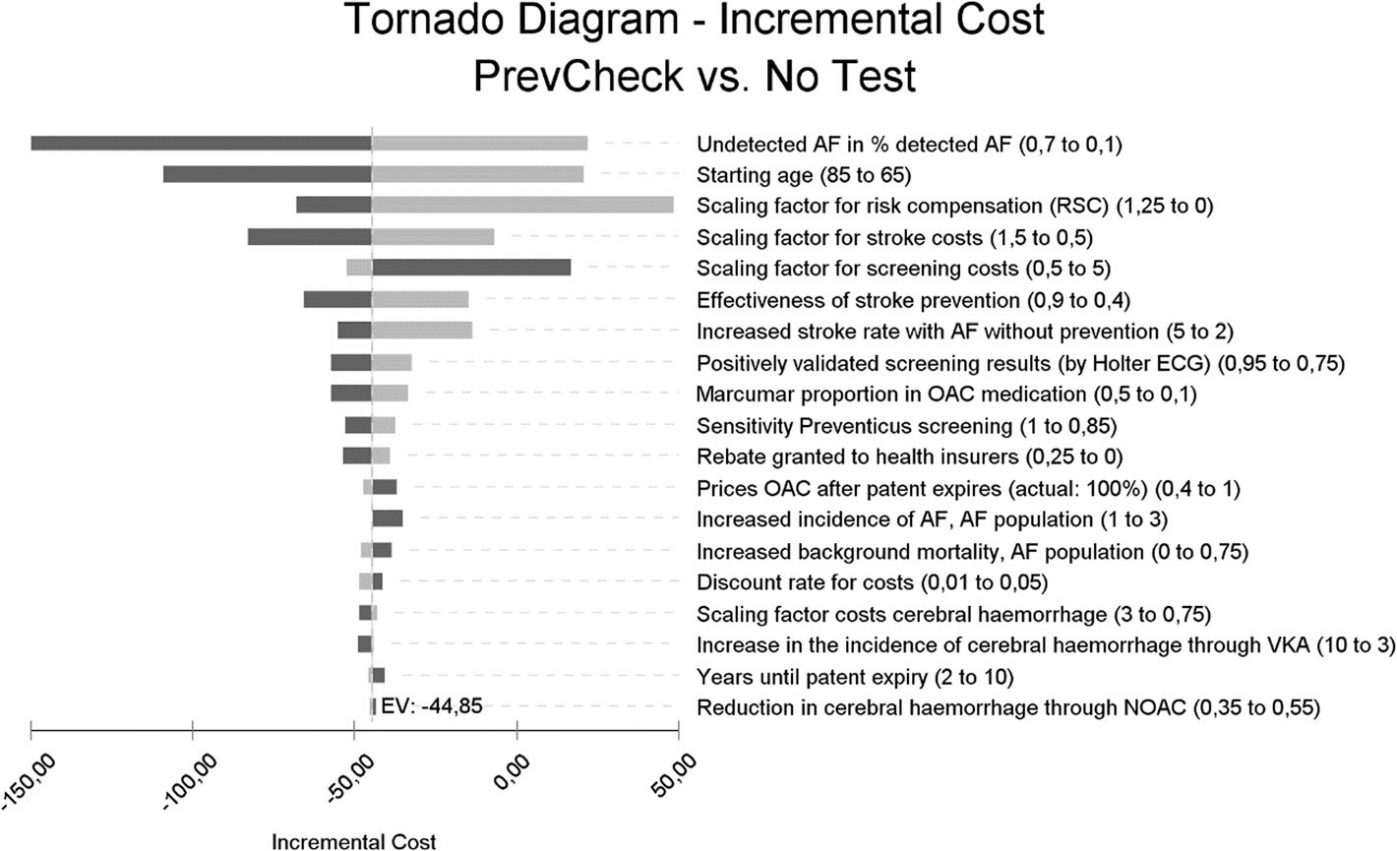
Tornado chart: effect of the isolated parameter changes of 19 model parameters on the model result (each ceteris paribus), timeframe: 4 years, men; base case: starting age 75 years old

Figure 6 shows the tornado chart for the timeframe 4 years for men with a starting age of 75. Compared to the timeframe remaining lifetime, the starting age and the scaling factor RSA gain in importance. The complete cessation of the RSA would result in a negative cost delta of €49 for men and €51 for women. Negative earnings effects also arise if extreme changes occur in other parameters. One example is a strong reduction of the “prevalence parameter” (undetected AF in % of detected AF) from 33 to 10% (see Table 2). As a result of the shorter timeframe, the effect of the discount factor is reduced. The parameter “undetected AF in % of detected AF” and the parameters linked to the frequency and the costs of a stroke, have a strong impact on the model result, even in the shorter timeframe.

**Table 2:** Results of the sensitivity analysis for 9 parameters with a high degree of fluctuation and the discount factor, timeframe four years, starting age 75 years old

Parameter	Variable		Minimum value	Delta costs ^a^	Maximum value	Delta Costs ^a^
Base case, model timeframe 4 years, starting age 75 years Discounted by 3%		M F		44.85 € 19.31 €		
pAF	Undetected AF in % of detected AF population (base case: 33%)	M F	10%	−21.91 € −29.57 €	70%	149.75 € 96.13 €
cStroke_ Factor	Scaling factor stroke costs 100% = base case	M F	50%	6.85 € −7.94 €	150%	82.84 € 46.57 €
RSA_Factor	Scaling factor RSA compensation payments; base case: factor 1.0	M F	0.0	−48.57 € −50.53 €	1.25	68.20 € 36.77 €
orStroke_AF_ reduction	Effectiveness of stroke prevention 0% - no effect; base case: 70%	M F	40%	14.87 € −2.51 €	90%	65.86 € 34.58 €
AgeStart	Starting age of the population Base case: 75 years old	M F	65 years old	−20.72 € −36.56 €	85 years old	109.05 € 71.69 €
orStroke_AF_ NOATT	Increased stroke rate with AF without prevention; Base case: factor 4.2	M F	2.0	13.81 € −3.48 €	5.0	55.05 € 26.85 €
cTest_Factor	Scaling factor screening costs 100% = base case (€47.54 / €297.50)	M F	50%	52.52 € 25.27 €	500%	−16.55 € −28.33 €
orMortAF_base	Increased background mortality, AF subpop. 25% increase = base case	M F	0%	48.17 € 20.99 €	75%	38.77 € 16.19 €
Discount rate	Discount factor 3% = base case	M F	1%	48.42 € 21.99 €	5%	41.51 € 16.82 €

*a Delta costs: cost advantage for screening strategy (if positive), cost disadvantage for screening strategy (if negative)*

Table 2 lists the results for nine parameters with a high degree of variation that appear in the shorter timeframe.

### Probabilistic sensitivity analysis (PSA)

#### Cost delta

**Fig. 7:**
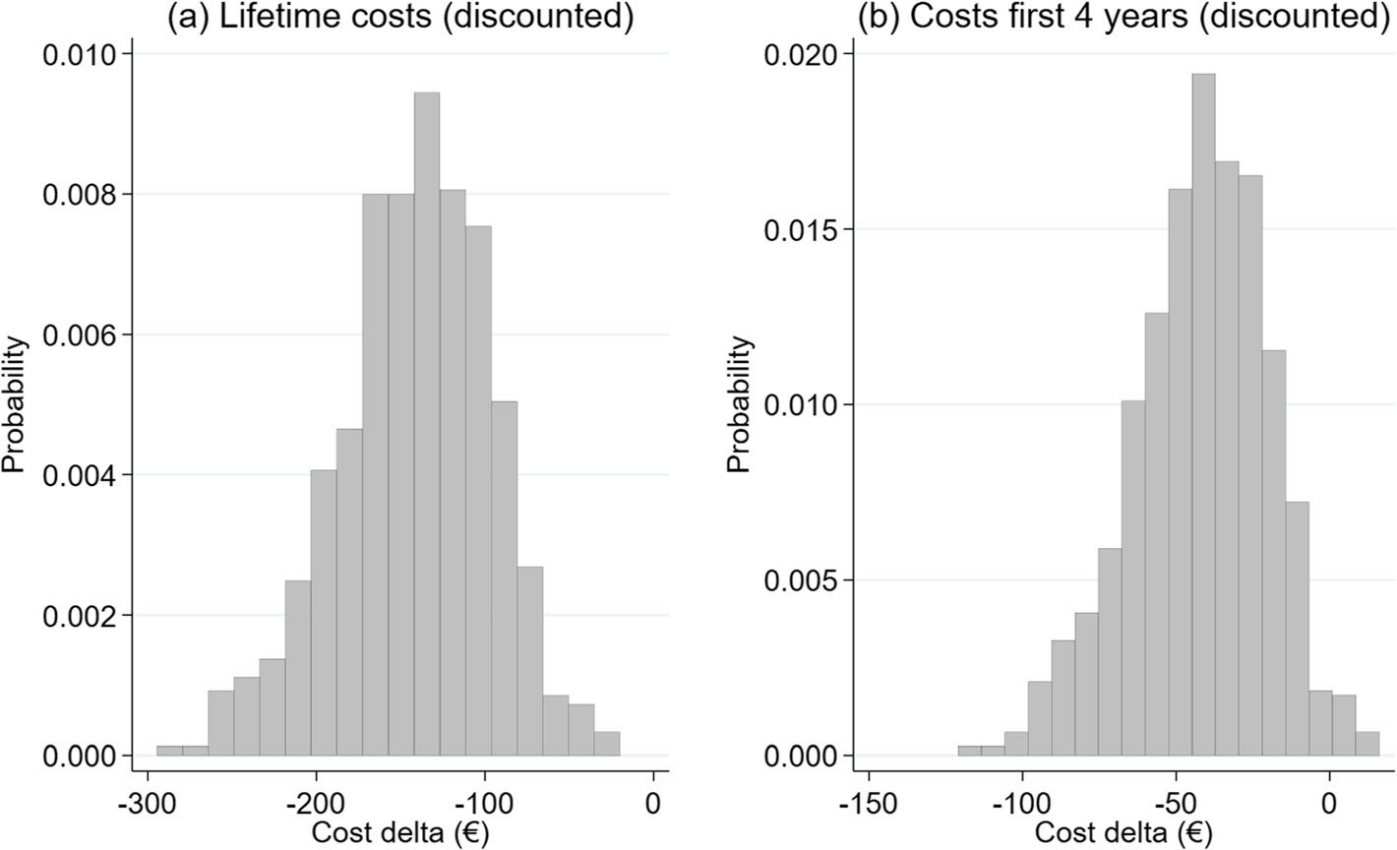
Result of the probabilistic sensitivity analysis: expected change in the result (Delta costs including RSA effect), timeframe lifetime (a), 4 years (b), men, base case: starting age 75 years old

The distribution of expected cost changes (including the RSA effect) is shown in Fig. 7 (a). The simulation shows the distribution for men, a timeframe lifetime and a starting age of 75. The values are scattered around an average value of -€109 (Base Case: -€147.67) with a standard deviation of €41. No cost increases were observed in any of the 1000 simulations. The values are scattered between -€258 (best value) and -€6 (worst value). With a 95% probability, the endpoint is situated between -€189 and -€29.

For a timeframe of 4 years, the distribution moves to the right. The distribution of the endpoint “Expected cost change” (including the RSA effect) is shown in Fig. 7 (b). The values are scattered around an average value of -€45 (base case: -€44.85) with a standard deviation of €21.7. The values are scattered between -€132 (best value) and + €24.5 (worst value). With a 95% probability, the endpoint is situated between -€91 and -€6.

#### Incremental cost-effectiveness analysis (ICER)

**Fig. 8:**
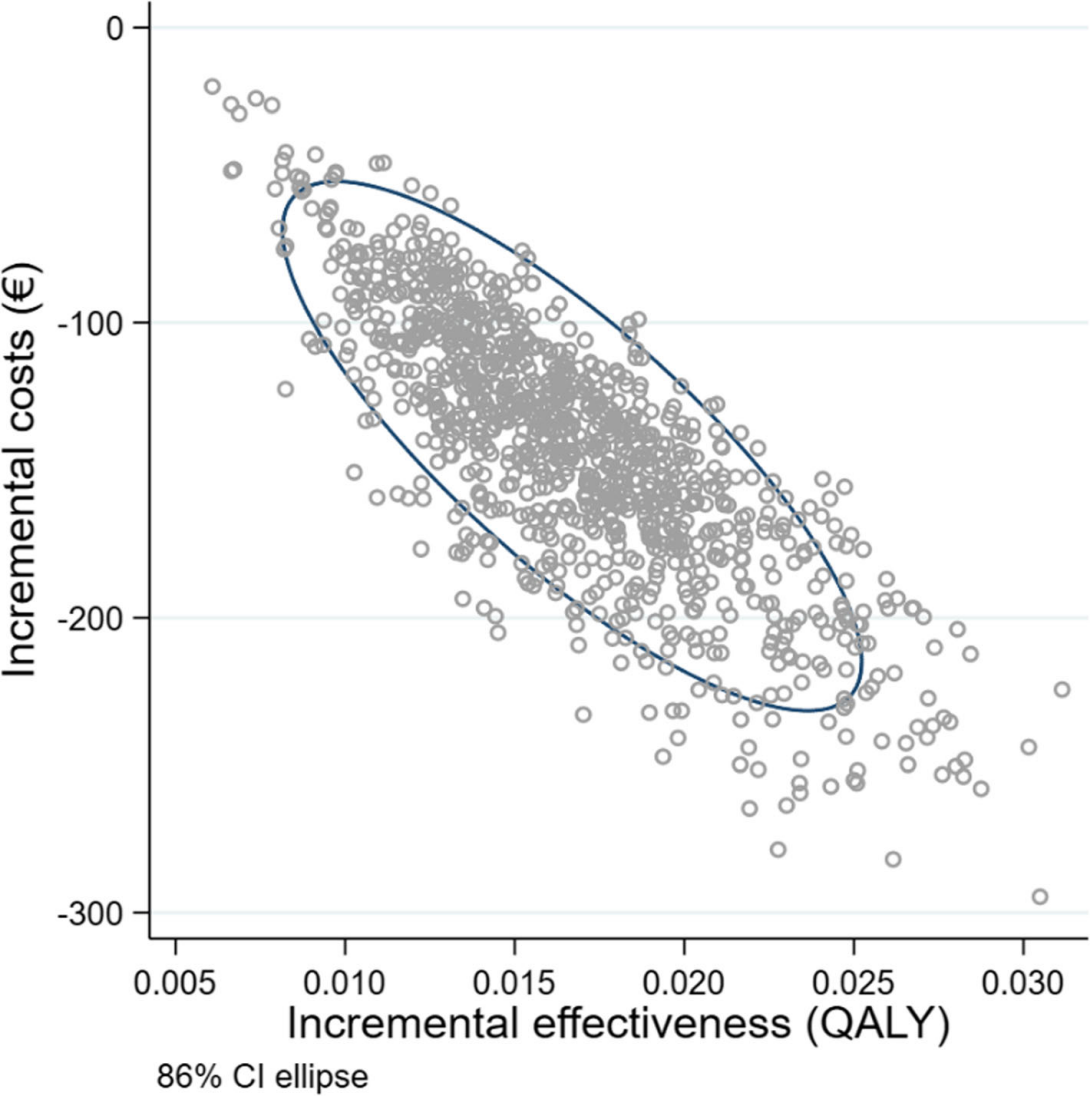
Result of the probabilistic sensitivity analysis: incremental cost-effectiveness and 95% confidence ellipse, timeframe lifetime, men; base case: starting age 75 years old

The result of the screening shows a slight increase of the quality-adjusted life years from 7.907 to 7.923 years (standard deviation 0.08 QALY) for men with a starting age of 75 and the timeframe remaining lifetime, whereby the costs have also decreased (taking into account the RSA effect). This means that, when it comes to choosing between the alternatives “Preventicus Heartbeats screening” and “no screening”, the alternative “Preventicus Heartbeats screening” is dominant (i.e. has a higher effectiveness with less costs). The result of the Incremental Cost-Effectiveness Analysis for men, starting age of 75, is shown as a two-dimensional ICER graph in Fig. 8.

The result for women, starting age 75 years old, corresponds to the result for men. The costs have decreased, and the number of quality-adjusted life years rises from 9.258 to 9.273 years (standard deviation 0.07 QALY).

## Discussion

The present model shows the cost-effectiveness of a systematic screening procedure for a population compared to the alternative (no screening). Although the screening goes hand in hand with higher costs for stroke prevention, the costs are already offset in the short term by the lower costs for acute treatment and follow-up treatment, which result from the lower number of strokes. Added to this are compensation amounts from the morbidity-oriented risk structure compensation scheme, which lead to a positive change to the results because of the screening.

Central cost data, such as the treatment costs for a stroke, were compiled from German sources. This is also the case for the incidence and prevalence data used and the restrictions for the mortality rates.

The literature lists a series of other cost-effectiveness models to assess procedures to detect previously undetected atrial fibrillation using a screening procedure. A systematic literature review and a model on the topic from a health economics point of view were carried out by [37] as part of a Health Technology Assessment for the NICE Institute. Another model of note is a cost-effectiveness model by the HIQA Institute in Ireland [38] as part of an HTA, which was also based on Irish healthcare information. The comprehensive model as part of the NICE-HTA showed an average approval rate (“uptake ratio”) of 64%. The average proportion of previously undiagnosed AF among the screening participants was 35%. Overall, systematic opportunistic strategies performed better than the systematic screening of entire populations and the cost-effectiveness increases with an increasing starting age ([37], Table 47).

With regard to the implementation of the screening, the Swedish “Strokestop” study [15] was found to be very similar to the “Preventicus Heartbeats” screening [36]. carried out a cost-effectiveness analysis based on the results and methods of the “Strokestop” study. At an increased cost of €50 per participant, the study showed an increase of the quality-adjusted life years from 6.646 to 6.657 QALY and an ICER ratio of €4313 per additional QALY. Compared to the reference scenario (Standard Care), eight cases of stroke were prevented among 1000 screening participants during the remaining lifetime of the simulated subjects.

For Switzerland, the cost effectiveness for the use of anticoagulant therapy was estimated between CHF 9702 and CHF 25,108 for the gain of a QALY [39].

The result of the studies mentioned is a cost increase due to the screening measures, which is accompanied, however, by an increase in effectiveness.

The presented cost-effectiveness model, on the other hand, showed a decrease in costs for almost all observed scenarios, accompanied by an increase in effectiveness. This effect is even greater because of the RSC compensation payments, which are not included in foreign studies, but which fully impact the results and premiums for statutory healthcare insurers in Germany.

A major difference compared to the “Strokestop” model [37] is the low screening costs (for the “Preventicus Heartbeats” scenario €47.54 for the app incl. Quality assurance and the examination of obvious defects in case of conspicuous results and €297.50 for the validation by means of a 14-day ECG), which only apply in case of positive and quality-tested app results [36]. lists costs of €108 for registration and ECG measurement in the 1st phase. In the second phase, the costs are €266 for a 24-h ECG.

The increased stroke rate in the presence of AF without prevention is three times the rate of that with prevention, according to [36], whereas it is four times the rate in this model and in the Framingham study [38, 40]. shows an age-dependency of the relative risk of stroke associated with AF in the Framingham data (Fig. 5.8). A scenario with threefold increased stroke risk was tested in our sensitivity analyses, without changing the basic statement of the present model.

As is the case in other studies (such as the Irish HTA by [36, 38] assume lower stroke costs. Aronsson et.al. [36] cite a Swedish study on stroke costs, which yielded €18,175 for the first year after the acute event and €4336 as costs for subsequent years. The 50% reduction in the costs of a stroke shown by this model (€21,060 in the first year and €6231 in subsequent years) were also tested in a sensitivity analysis (see Tables 1 and 2).

The differences between the design of the model used in this study and other cost-effectiveness models offer starting points for optional further development. The presented model regards age and gender as significant influential factors for the incidence rates [36], for example, use the CHA_2_DS_2_VASC score of the 1000 simulated participants of the Strokestop study. The Markov model in the NICE-HTA [37] includes health states which summarise different previous events (e.g. bleedings and strokes). This model only assigns the simulated subjects one health state each at any given point in time. Also different from other studies is the number of simulated phases per year in the remaining lifetime. The length of the phases in this study is 1 year, whereas other studies include several multi-year phases.

A starting age of under 65 years is not included in the model. If the participant cohort is limited to individuals with an increased CHA_2_DS_2_VASC score, an improved model result can also be expected for younger participants. To quantify this effect, this model approach should be expanded by including the CHA_2_DS_2_VASC score, which can be based on study results for younger arrhythmia patients and their risk factors, insofar as these are available. From the viewpoint from comparing the reliability to the simulation results, the future study had better take a heterogeneous population like Herman et al. [41] into account.

## Conclusions

The present study shows the positive effect on the results and premiums of a systematic screening by means of the Preventicus screening procedure using population and cost information which is relevant for the implementation of the screening procedure in Germany.

## Data Availability

All data generated or analyzed for the economic model are included in this published article.

## Appendix: Model parameters

**Share of undetected AF in the screening population:** The model assumption based on the findings of the “Strokestop” study [15] and a systematic review [42]. In the base case, it is assumed that the proportion of participants with undetected AF is 33% of the prevalence of known AF in the screening population. The AF prevalence values are based on the findings of a study by [43] that analysed data from Barmer GEK [German health insurer]. The percentage of participants with undetected AF is linked to the prevalence of AF and therefore dependent on the starting age and gender of the screening cohort.

**Sensitivity and specificity** of the “Preventicus Heartbeats” screening. The values (sensitivity: 91.5%, specificity: 99.6%) are based on [31]‘s study. It is assumed that 85% of cases detected by the “Preventicus Heartbeats” screening are confirmed with a Holter ECG in the two-week validation period [44].

**Starting age of the cohort:** Analogous to the “Strokestop” study, a starting age of 75 was chosen in the Base Case. Gender and starting age were provided for a simulated cohort. General (“background”) mortality was chosen from the German mortality tables [45]). The stroke mortality was deduced from the background mortality based on standardised mortality rates [46].

**Incidence and prevalence:** The following incidence and prevalence rates are dependent on age and gender:

- Incidence and prevalence of atrial fibrillation [43] with an impact on the prevalence of undetected AF in the screening population
- Incidence of ischaemic strokes ([3], published in age ranges of 10 years, exponentially interpolated)
- Incidence of intracranial haemorrhages ([47], published in age ranges of 20 years, exponentially interpolated).

The incidence rates for AF, stroke and intracranial haemorrhage are based on normal populations. For patients who suffer from AF and in case of prevention with anticoagulants, the incidence and prevalence rates are higher.

The transition probabilities are essentially based on the table values for mortality and incidence and are therefore age- and gender-dependent. For some parameters (e.g. haemorrhages) probabilities are chosen that are independent of age and gender. The following events can be distinguished: Stroke, intracranial haemorrhage, other bleeding and mortality. Age- and gender dependent parameter values are listed in Table 4.

**Stroke:** Assumptions are based on the publication by [3]. The probability depends on the fact whether AF is present and - if so - whether there is stroke prevention:

- AF is not present: use of interpolated original values (factor 1)
- AF is present, no stroke prevention with OAC: the original values are multiplied by a factor of 4.2 (result of the Framingham Study, [40]). To prevent calculated probabilities greater than 1, the factor is interpreted as an odds ratio.
- AF is present, stroke prevention with OAC: according to studies on the effectiveness of vitamin K antagonists and the new oral anticoagulants. Meta analyses comparing Warfarin against Placebo [23] and Warfarin against direct OAC [26] suggest a total stroke reduction of 70 to 80%. We assume a decrease of around 70% compared to cases without prevention with OAC.
- For the subsequent years after the initial stroke, a recurrence rate of 12% p.a. is assumed for a new stroke. This number is based on the results of [48] and [49], who obtained a five-year recurrence rate of 24% for the Erlangen register.

**Intracranial haemorrhage (ICH):** The probability is based on the statistics of [47]. If AF is present, this is adjusted. This is also the case for prevention with OAC.

- AF is not present: use of the original values of [47].
- AF is present, no stroke prevention with OAC: Multiplication of the original values by factor 4. The assumption is based on the increased number of ICH, reported by a Swedish cohort study [50].
- AF is present, stroke prevention with OAC: in cases treated with vitamin K antagonists, no further increase is assumed compared to cases without prevention. If treated with NOAC, the probability of suffering an intracranial haemorrhage is reduced by a factor of 0.41. This is based on the results of the NOAC-Warfarin-comparisons included in the meta-analysis by [51].

**Other bleeding:** Under certain conditions, atrial fibrillation is linked to other types of bleeding. The model distinguishes:

- Severe bleeding (“major bleedings”, e.g. gastrointestinal bleeding). For participants with AF who do not receive stroke prevention, a probability of 2.3% is assumed (source [50], Table 6). This probability increases to 3.9% in cases of stroke prevention (value averaged from 5.2% in cases of prevention with vitamin K antagonists and 3.3% in cases of prevention with NOAC (source [29], Table 3). It could be considered that it is reported that some NOACs may increase gastrointestinal bleeding
- Less severe bleeding (“minor bleedings”): a study on the NOAC Dabigatran determined a 14% probability for less severe bleeding in case of stroke prevention with Dabigatran ([29], Table 3). For the probability of less severe bleeding when AF is present with no stroke prevention, it is assumed that the value corresponds to approximately 6/10 of the bleeding probability with OAC medication, as is the case with severe bleeding (result: assumption of 8.3%).
  Once the bleeding has been treated, the OAC medication is continued in accordance with the AF guidelines [25].

**Table 3:** PSA distributions and parameters, base case values

Original name	Type	Param 1	Param 2	Base Case	Description
**General**					
Undetected AF in % detected AF	Beta	37.9	962.1	0.038	Assumption (prevention, 1/3 ratio undetected AF to detected AF)
Sensitivity Preventicus screening ^b^	Beta	229	21	0.92	from DETECT AF [31]
Specificity Preventicus screening ^b^	Beta	342.624	1.376	0.99	from DETECT AF [31]
Positively validated screening results (after Holter ECG) ^b^	Beta	9.9875	1.7625	0.85	Adoption acc. to Wachter et.al [44].
Prevention					
Marcumar proportion in OAC medication	Triangular	0.05 / 0.2 / 0.5		0.29	
Increased stroke rate with AF without prevention	Normal	4.2	0.235	4.20	mean: Wolf et.al [40]. SD: assumption
Reduction of stroke rate through prevention ^b^	Normal	0.686	0.05	0.70	Assumptions, based on Hart [23] and López-López [26]
Strokes and Mortality					
Stroke rate normal population	Normal	1.00	0.14	1.00	SD according to Kolominisky-Rabas [49]
Frequency of recurrent stroke	Beta	52.48	275.52	0.160	Hardie [48]: Perth registry
Mortality, year 1 (SMR)^a^	Normal	3.7	0.3	3.7	Bromum-Hansen [46], year 0–1
Mortality, subsequent years (SMR) ^a^	Normal	1.92	0.13	1.92	Bronnum-Hansen [46], years 2–5
Mortality, recurrence (SMR factor)	Triangular	1.0 / 1.5 / 2.0		1.5	Assumption according to Hardie [48]
Stroke costs, year 1	Gamma	434.0277	0.02344	18,517	Kolominsky-Rabas [3] / variation coefficient derived from lifetime, 2006 prices
Stroke costs, subsequent years	Gamma	434.0277	0.07922	5479	discounted costs, 2006 prices
Increased mortality, AF subpopulations	Normal	25&	5%	25%	Wolf, Mitchel [52]
Frequency of bleeding					
Severe bleedings, without OAC:	Normal	0.023	0.00153	0.023	Friberg [29, 50], Granger: Apixaba n[29]
Severe bleedings, with OAC	Normal	0.039	0.0026	0.039	Mix of VKA and NOAC
Less severe bleedings, without OAC	Normal	0.08256	0.00242	0.0823	Assumption: ratio with/without same as with severe bleeding
Less severe bleedings, with OAC	Normal	0.14	0.0041	0.14	Krejczy [53],Grange r[29],
Cerebral haemorrhages					
Increase in the incidence of brain haemorrhage through VKA	Normal	4.00	0.22	4.00	Assumption, SD analogous to Wolf [40]
Reduction in cerebral haemorrhages through NOAC (compared with VKA)	Beta	24.34211	35.02890	0.41	Chatterjee [51], suppl., eFigure 3
Mortality cerebral haemorrhages	Beta	37.422	39.578	0.486	Fang [54]
QALYs					
Age decrement (per year)	Normal	−0.00029	0.0000225	−0.00029	Sullivan [55]
with AF	Beta	33.82	7.93	0.810	Gauthier: HTA Canada [56] citng Sullivan et.al [57, 58]
with AF: reduction factor with VKA medication ^b^	Uniform	0.953	1.000	0.987	Shah & Gage [59] (range), Gage et.al [60].
with AF: reduction factor with NOAC medication ^b^	Uniform	0.990	0.998	0.994	Shah & Gage [59] (range), O’Brien, Gage [61]
after cerebral haemorrhage, year 1	Beta	11.41332	17.19149	0.399	Golicki [62] (ICD I61 result)
after stroke, year 1	Beta	378.6275	304.81567	0.554	Golicki [62] (ICD I63 result)
after stroke: reduction in the year of the event	Normal	0.103	0.008	0.103	Gauthier: HTA Canada [56], citing Sullivan et.al [57].
after stroke or cerebral haemor- rhage, subsequent years ^b^	Uniform	0	0.5	0.12	Shah & Gage [59] (range)
Decrement for severe bleeding	Normal	−0,092	0.010	−0,092	Gauthier: HTA Canada [56], Freeman [63], Thomson [64]
Decrement for less severe bleeding	Normal	−0.013	0.001	−0.013	Gauthier: HTA Canada [56], Freeman [63], O’Brien [61]

^a^ Values for men (75 years; if age-dependent) ^b^ Different average in EV (expected value) calculation

Parameters: by distribution type (Param1, Param2): Beta distribution (alpha, beta), Gamma distribution (alpha, lambda), Normal distribution (mean, standard deviation), Uniform distribuiton (min,max), Triangular distribution (min/ likeleliest/max)

**Mortality.** Following the results of Wolf [52] and Friberg [65], background mortality is adjusted for the subpopulation with AF. The increased mortality is partly due to the increased risk of stroke, but also due to other cardiovascular diseases like heart failure and ischaemic hart diseases ([65], Table 3). In the base case model, background mortality for the AF subpopulations will be increased by 25% (assumption, based on the differences in mortality occurrence shown by [52], Table 2).

A distinction is made between mortality with and without stroke:

- Mortality without stroke: this is represented in the mortality tables [45].
- Mortality after stroke: use of the “standardised mortality rates” of stroke patients, collected in Denmark [46]. A distinction is made between mortality in the first year after the stroke and in subsequent years.
- Mortality after a second stroke: increase of 50% over the “standardised mortality rates” after the initial stroke. The assumption is based on figures from the Perth register [48], which reported a 22% mortality rate for the first 28 days after the initial event and a 41% mortality rate after the subsequent event.
- Mortality after intracranial haemorrhage: 48.6%; source [54], Fig. 1.

**Later detection of AF:** For participants with undetected AF or non-existing AF, the incidence figures collected from [43] were used as probabilities for the later detection or the spontaneous occurrence of atrial fibrillation. This is true for both the “Preventicus Heartbeats” scenario and the scenario without screening.

**Stroke prevention with oral anticoagulants:** All patients who were found to suffer from AF in the screening or at a later point in time received a treatment for stroke prevention. The following assumptions are made:

- The proportion of vitamin K antagonists (usually Phenprocoumon/Marcumar®) in the treatment is 29% (in the base case). This is an assumption, as no figures have been published on the use of oral anticoagulants for AF diagnosis in Germany. According to [66] (Central Illustration), a 37.8% proportion of vitamin K antagonists was determined in the GLORIA-AF project. The lower value used in the model is based on the fact, among other things, that stroke prevention is no longer carried out with VKA but with NOAC for almost all new cases.
- The adherence to drug therapy is assumed to be initially 100%. After the first year, 10% of patients stop the medication and in subsequent years 5% of the remaining patients given they have not changed to a different state of health. These changes are all based on assumptions.

The model considers cost and earnings components for the model sections screening, stroke prevention, acute stroke and cerebral haemorrhage and the costs they create, and the bleedings that occur as side effects of the preventive treatment with oral anticoagulants. Atrial fibrillation, stroke and cerebral haemorrhage trigger compensation payments in the current catalogue of the morbidity-oriented risk structure compensation scheme, which are (optionally) included in the model as earnings (see Table 5).

**Screening:** All participants of the screening (“Preventicus Heartbeats” scenario) firstly incur costs for the use of the “Preventicus Heartbeats” app. A sum of €47.54 including 19% VAT is fixed in the base case as usage costs. The subsequent validation during a two-weekly measurement by means of a continuously measuring and telemetric Holter ECG and final assessment and diagnosis by a cardiologist costs €297.50 including 19% VAT in the base case.

**Stroke prevention with oral anticoagulants:** the costs are a combined amount from the treatment with vitamin K antagonists (VKA, Marcumar®, daily cost of €0.20) and NOAC (new oral anticoagulants: Apixaban, Dabigatran, Rivaroxaban, Edoxaban, simplified assumption for the daily costs: €3.00). A Marcumar® share of 29% is assumed, which corresponds to average annual costs (before discounts) of €800. There is only approximate information available about the **rebates** currently granted to health insurers. A rebate of 10% is used in the base case.

For the **time after the NOAC patents expire** (assumption: within 3 years after the start of the model), the model assumes that the NOAC costs decrease to 55% of the current level. This assumption is varied in the sensitivity calculations.

For stroke prevention, costs are incurred for **out-patient treatment**. Out-patient treatments are assumed in all four quarters, both for prevention with NOAC and for Prevention with VKA (fixed doctor’s fee per quarter according to EBM catalogue number 0300: €16.73 for insured persons up to 75 years old, €22.37 for insured persons over 75 years old). Insured persons undergoing treatment with Phenprocoumon/Marcumar® also incur a cost of €0.75 for the monthly measurement of the coagulation status (EBM catalogue no. 32110) [67].

**Stroke costs:** [3] created a model on the basis of the Erlangen register [68] of the lifetime costs of a stroke, taking into account the development of the German population between 2005 and 2025. The average lifetime cost amounted to **€43,129**, mainly determined by the outpatient part. The total cost amounted to €18,517 in the first year and €5497 in subsequent years. Due to the temporal distance from index year 2006, higher costs must be assumed. Based on the consumer price index for healthcare costs [69] a cost increase of 13.7% must be assumed for the period 2006–2018. The total cost of the base case amounts to €21,060 in the first year and €6231 in subsequent years.

**Cost of intracranial haemorrhage (ICH):** we were not able to identify any study that quantifies the costs for the German healthcare system. DRG statistics of the Institute für Entgeltwesen im Gesundheitswesen ([70] [Institute for compensation in healthcare]) show that, when comparing the ICD10 Figs. I61 (hemorrhagic strokes) and I63 (ischemic strokes), the case-by-case hospital costs for cerebral bleeding is 1.8 times as much as the case-by-case costs of ischemic strokes. For this model, 1.5 times the case-by-case cost of an ischaemic stroke is used. The assumption is varied in a sensitivity analysis.

**Cost of bleedings**

- Severe bleedings: an average cost rate from the DRG costs for gastrointestinal bleeding (DRG catalogue numbers G70A-C and G46B) is used. This amounts to a cost of €2025 per bleeding.
- Less severe bleedings: to simplify, the cost assumption by [53] was adopted for the amount of €50 per bleeding.

**Refunds as part of the risk structure compensation scheme:** the reimbursement amounts incurred as part of the morbidity-oriented risk structure compensation scheme, are published by the German Federal Insurance Office. This is based on the reimbursement amounts for compensation year 2017 [71]. It is assumed that the three morbidity groups concerned will also remain part of the risk structure compensation scheme in the long term. The morbidity groups concerned are:

- Atrial fibrillation (homogeneous morbidity group HMG 092): the compensation amount is €1249 for in-patient treatment or treatment for at least two quarters. It is assumed that both prevention with VKA and prevention with NOAC are reimbursed. For the time after the NOAC patent has expired, it is assumed that the compensation amount decreases annually by 10%, until the reimbursement level before NOAC was implemented has been reached (approx. 700 euros before 2010).
- Strokes (HMG 096): €2248, for which a main hospital diagnosis is required. This should always be the case for acute or subsequent strokes.
- Cerebral haemorrhage (HMG 095): €5831. The requirements are the same as those for a stroke.
- The costs are discounted by 3% per year.

**Quality of life:** To assess the effectiveness of the measure, the model uses the change in quality-adjusted life years (QALY). QALYs reflect the state of health in each year, using Value 1 for full health and Value 0 for death (see e.g. [35]). The following assumptions are made for the model:

- QALY in the presence of arrhythmia: 0.81 (source: [56], citing [57]. QALY decrement based on the EQ-5D median scores for cardiac arrhythmias [58])
- QALY decrements for aging have been estimated by [55] (Fig. 1). The base case assumes a QALY decrement of 0.00029 per year exceeding the age of 65 years.
- Drop in arrhythmia QALYs through stroke prevention: use of factor 0.987 for VKA treatment (Source: [59, 60] and 0.994 for NOAC treatment on the arrhythmia QALY (source: [59, 61])
- QALY after initial stroke event: 0.55 (source: [62])
- QALY after subsequent stroke event: 0.12 (source: [59])
- QALY after intracranial haemorrhage, initial event: 0.399 (source: [62]) Subsequent events are evaluated as for strokes.
- QALY decrements for bleedings: in cases of severe bleeding, 0.092 is deducted in the year of the bleeding (sources: [56, 63, 64]). In cases of mild bleeding the decrement is 0.013 in the year of the bleeding (sources: [56, 61, 63])
- QALYs are discounted by 3% per year.

Tables 3 through 6 summarize the model parameters. Table 3 lists the base case parameter values and PSA distribution assumptions. Table 4 lists the age- and gender-dependent parameter values. Table 5 summarizes the unit cost parameters of the model and Table 6 lists the parameters which have been examined in the deterministic sensitivity analyses.

**Table 4:** Age- and gender-dependent parameter values

Life years	Gender	Back-ground mortality	Stroke mortality, year 1	Stroke mortality, subse-quent years	AF incidence	AF preval-ence	Undetect- ed AF (preval-ence)	Incidence of strokes	Incidence of intra-cranial haemor-rhages
65	M	1.54%	5.46%	2.91%	0.99%	5.00%	1.67%	0.42%	0.04%
	F	0.80%	4.02%	1.63%	0.64%	2.72%	0.91%	0.27%	0.02%
70	M	2.24%	7.82%	4.21%	1.39%	7.80%	2.60%	0.63%	0.06%
	F	1.22%	6.01%	2.47%	1.06%	4.97%	1.66%	0.45%	0.04%
75	M	3.40%	11.53%	6.34%	1.89%	11.37%	3.79%	1.03%	0.10%
	F	1.91%	9.15%	3.83%	1.58%	8.09%	2.70%	0.99%	0.07%
80	M	5.86%	18.72%	10.67%	2.39%	14.57%	4.86%	1.36%	0.17%
	F	3.84%	17.15%	7.57%	2.14%	11.29%	3.77%	1.26%	0.12%
85	M	10.75%	30.83%	18.79%	3.01%	16.92%	5.64%	2.42%	0.29%
	F	7.72%	30.24%	14.64%	2.77%	13.42%	4.47%	2.01%	0.21%

**Table 5:** Cost parameters

Parameter	Value (€)	Comment
**Preventicus screening**		
Preventicus app	47.54	
Assessment and diagnosis	297.50	
**Outpatient treatment**		Source: EBM catalogue [67]
GP consultation, age below 76	16.73	EBM catalogue no. 0300
GP consultation, age 76 or above	22.37	
INR coagulation measurement	0.75	EBM catalogue no. 32110
**Medication**: VKA	0.20	Yearly costs: 73 €
Medication: NOAC	3.00	Yearly costs: 1095 €
NOAC prices after patent expiration (% of current prices)	55%	Reduced yearly costs: 602 €
Medication: rebate granted	10%	Assumption
**Strokes / ICH**		
Stroke costs, year 1	21.060	2018 costs, inflated data based on
Stroke costs, follow-up years	6.211	Kolominsky-Rabas [3]
Inflation factor 2006–2018	113.7%	Statistisches Bundesamt
ICH, percentage of stroke costs	150%	Assumption
**Bleeding**		
Minor bleeding	50	cost assumption by [44]
Major bleeing	2.050	DRG catalogue numbers G70A-C and G46B)
**Risk structure compensation**		Source: 2018 HMG catalogue values
HMG 092 (Arrhythmias)	1.249	
HMG 092 decrease after NOAC patent expiration	90%	per year. Assumption: compensation will bedecreased because of lower medication costs until a minimum of 700€ is reached.
HMG 096 (Ischaemic strokes)	2.248	
HMG 095 (ICH)	5.831

**Table 6:** Parameters of the deterministic sensitivity analysis

	Sensitivity analysis		Base case
Model parameters	min	max	
**General** Starting age	65	85	75
Undetected AF in % detected AF	10.0%	70.0%	33.3%
Increase in background mortality, AF subpopulation	0%	75.0%	25.0%
Sensitivity Preventicus screening	85.0%	100.0%	91.6%
Specificity Preventicus screening	85.0%	100.0%	99.6%
Positively validated screening results (after flutter ECG)	75.0%	95.0%	35%
Increased incidence of spontaneous AF, AF population	1.0	3.0	1.0
**Prevention** Reduction stroke rate with prevention	40.0%	90.0%	70.0%
Increased stroke rate with AF without prevention	2.0	5.0	4.2
Marcumar proportion in OAC medication	10.0%	50.0%	29.0%
Increase in the incidence of brain haemorrhage through VKA	3.0	10.0	4.0
Reduction in cerebral haemorrhage through NOAC (compared with VKA)	35.0%	55.0%	41.0%
**Costs, profits**			
Scaling factor screening costs	0.5	5.0	1.0
Prices OAC after patent expires (actual: 100%)	40%	100%	55%
Clawback (rebate granted to health insurers?)	0.0%	25.0%	10.0%
Years until patent expiry	2	10	3
Scaling factor stroke 1- costs	0.5	1.5	1.0
Scaling factor costs cerebral haemorrhage	0.75	3.0	1.0
Scaling factor RSA	0.0	1.25	1.0
Discount rate for costs	1.0%	5.0%	3.0%

## Abbreviations

AF: Atrial Fibrillation
ATT, NOATT: Anti-thrombotic treatment resp. No anti-thrombotic treatment
CI: Confidence interval
DRG: Diagnosis related groups
EBM: Unified standard of evaluation, German reimbursement catalogue, (German: *Einheitlicher Bewertungsmaßstab*)
ECG: Electrocardiogram
EV: Expected value
HIQA: Health Information and quality authority (Ireland)
HMG: Homogeneous morbidity groups
HTA: Health technology assessment
ICD: International classification of diseases
ICER: Incremental cost-effectiveness ratio
ICH: Intracerebral hemorrhage
NICE: National institute for health and care excellence (England)
NOAC: Novel (direct) oral anticoagulants
OAC: Oral anticoagulants
QALY: Quality adjusted life years
RSC/RSA: Risk structure compensation scheme /(German: *Risikostrukturausgleich*)
SD, Std. Dev.: Standard deviation
SGB: German social act (German: *Sozialgesetzbuch*)
VAT: Value added tax
VKA: Vitamin K antagonists
